# Homocysteine, methylenetetrahydrofolate reductase C677T polymorphism, and risk of retinal vein occlusion: an updated meta-analysis

**DOI:** 10.1186/1471-2415-14-147

**Published:** 2014-11-27

**Authors:** Dan Li, Minwen Zhou, Xiaoyan Peng, Huiyu Sun

**Affiliations:** Department of Ophthalmology, Beijing Institute of Ophthalmology, Beijing Ophthalmology and Visual Science Key Lab, Beijing Tongren Eye Center, Beijing Tongren Hospital, Capital Medical University, 17 Hougou Lane, Chongnei Street, Beijing, 100005 China; Department of Ophthalmology, Beijng di tan hospital, Capital Medical University, Beijing, China; Department of Ophthalmology, Shanghai First People’s Hospital, School of Medicine, Shanghai JiaoTong University, 100 Haining Road, Shanghai, 200080 P.R. China

**Keywords:** Homocysteine, Methylenetetrahydrofolate reductase, Retinal vein occlusion

## Abstract

**Background:**

To evaluate the role of plasma total homocysteine (tHcy) and homozygosity for the thermolabile variant of the methylenetetrahydrofolate reductase (MTHFR) C677T genotype in the risk of retinal vein occlusion (RVO).

**Methods:**

Relevant studies were selected through an extensive search of PubMed, EMBASE, and the Web of Science databases. Summary weighted mean differences (WMDs) or odds ratios (ORs) and 95% confidence intervals (CI) were calculated with a random-effects model.

**Results:**

Forty-two studies with 6445 participants were included in this updated systematic review and meta-analysis. The mean plasma tHcy level in the RVO patients was significantly higher than in the controls (WMD =2.13 μmol/L; 95% CI: 1.29 to 2.98, *P <* 0.001), but there was evidence of between-study heterogeneity (*P <* 0.001). No significant association between MTHFR C677T genotype and RVO was found under all genetic models.

**Conclusion:**

There was some evidence that plasma tHcy is associated with an increased risk of RVO. There was no evidence to suggest an association between homozygosity for the MTHFR C677T genotype and RVO.

## Background

Retinal vein occlusion (RVO) is one of the most common vision-threatening retinal vascular diseases, affecting males and females almost equally and occurring most frequently in elderly subjects [1, 2]. It is a multifactorial disease, which may affect small, medium, and large ocular vessels, with central occlusion representing the most dangerous clinical entity. Central retinal vein occlusion (CRVO) and branch retinal vein occlusion are the most common and clinically relevant types of venous occlusions. Arterial hypertension, diabetes mellitus, cigarette smoking, atherosclerosis, and increased plasma lipoprotein (a) have been reported as systemic risk factors for RVO [3–7].

Homocysteine (Hcy), a sulfur-containing amino acid formed during the metabolism of methionine, can be remethylated to methionine throughmethyltetrahydrofolate reductase (MTHFR) [8]. Several studies have shown that the level of plasma total homocysteine (tHcy) is elevated in RVO patients and it is a risk factor for RVO [9, 10]. The MTHFR C677T gene mutation is an important cause of elevated plasma tHcy. The mutation results in Hcy not being remethylated to methionine, leading to hyperhomocysteinemia [11, 12]. Although a number of studies have reported a correlation between the MTHFR C677T mutation and RVO, the role of the mutation in the pathogenesis of RVO remains unclear [13, 14].

A previous meta-analysis of 25 case–control studies conducted in 2009 showed that elevated tHcy was associated with RVO but not for the MTHFR C677T genotype [15]. However, this meta-analysis had some limitations, including a lack of information on the dose-effect relationship between tHcy and RVO. Another meta-analysis on the association of tHcy with RVO published in 2003 included only 19 case–control studies [16]. Since the meta-analysis was published, a variety of studies aimed at elucidating this relationship has yielded inconsistent results [10, 14, 17–20].

In the present study, we analyzed the relation among tHcy, the MTHFR C677T genotype, and RVO in an updated meta-analysis of case–control studies. The aim of this updated analysis of 42 studies was to derive a more precise estimation of the relationship among tHcy, the MTHFR C677T genotype, and the risk of RVO.

## Methods

### Literature search

A systematic literature search of PubMed, ISI Web of Science, and EMBASE was performed to identify relevant studies from inception until March 10, 2014. The following terms were used in the searches: “retinal vein occlusion” AND (“homocysteine” OR “methyltetrahydrofolate reductase”). The websites of professional associations and Google Scholar were also searched for additional information. When relevant articles were identified, their reference lists were searched for additional articles. The final search was carried out on March 10, 2014, without restrictions regarding publication year, language, or methodological filter.

### Inclusion and exclusion criteria

Studies included in this meta-analysis met the following criteria. The studies (a) contained a laboratory assessment of plasma tHcy concentrations or reported odds ratio (ORs) and their 95% confidence intervals (CIs) of the association between tHcy and RVO, or they assessed the MTHFR C677T polymorphism. Alternatively, (b) articles were retrieved if they were retrospective, prospective, or case–control studies. If multiple publications from the same study population were available, the most recent study would be eligible for inclusion in the meta-analysis. Editorials, letters to the editor, review articles, case reports, meeting abstracts, and animal experimental studies were excluded.

### Data extraction

Two authors (Z.M.W. and X.Y.P.) independently extracted the following data from the included studies: publication data (author, year of publication, and country of the population studied); patient condition (fasting status); participant’s age and sex; number of cases and controls; the Hcy levels in the cases and the control subjects; the adjusted ORs of the association between tHcy and RVO; and the genotype counts.

### Assessment of the quality of the methodology

Two reviewers independently assessed the quality of each study using the Newcastle-Ottawa Scale (NOS) [21]. The NOS uses a “star” rating system to judge quality based on three aspects of the study: selection, comparability, and exposure. The scores ranged from 0 stars (worst) to 9 stars (best). Studies with a score of ≥7 were considered of high quality [22, 23]. Any discrepancies were addressed by a joint re-evaluation of the original article with a third reviewer (D. L).

### Statistical analysis

The weighted mean differences (WMDs) were used to compare the plasma tHcy concentrations between the case and control subjects. The pooled adjusted ORs with their corresponding 95% CIs were used as a common measure of the association between tHcy and the risk of RVO. ORs and 95% CIs were calculated for the MTHFR C677T TT genotype exposure and RVO. The association between MTHFR C677T genotype exposure and RVO was examined using the following genetic models: the homozygote co-dominant (TT vs. CC), heterozygote co-dominant (TC vs. CC), dominant genetic (TT/TC vs. CC), and recessive genetic (TT vs. TC/CC) models.

We combined the data using a random effects model to achieve more conservative estimates [24]. Statistical heterogeneity between the studies was evaluated using Cochran’s Q test and the I^2^ statistic. For the Q statistic, *P* < 0.05 was considered to indicate statistically significant heterogeneity. A meta-regression analysis was used to investigate the influence of the variables on the study heterogeneity across strata. To detect publication biases, we calculated Begg’s and Egger’s measures [25, 26]. A *P* value less than 0.05 was considered statistically significant in the test for the overall effect. The analysis was conducted using the Stata software package (Version 12.0; Stata Corp., College Station, TX).

### Sensitivity analysis

A subgroup analysis was used to investigate which factors (diagnosis, sources of controls, adjusting factors, and overnight fasting status) might contribute to heterogeneity. Furthermore, we performed a sensitivity analysis by excluding the low-quality studies and reanalyzing the pooled estimate for the remaining studies.

## Results

### Literature search

**Figure 1:**
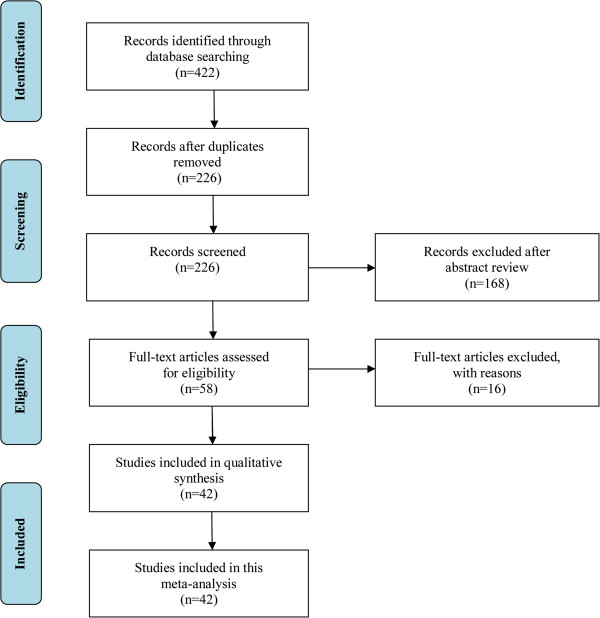
**Flow diagram outlining the selection process for the inclusion of the studies in the systematic review and meta-analysis.**

The literature search identified 422 papers. Of these, 196 were excluded because they were duplicate studies. Initially, the title, abstract, and medical subject heading words of the obtained publications were used for a rough judgment on the eligibility of an article. In total, 168 studies, including reviews and case series, were excluded for various reasons, such as being irrelevant to our analysis. The remaining 58 were retrieved for a full-text review. In total, 16 articles were excluded for various reasons. Of these, seven articles were excluded because they provided no data on plasma tHcy concentrations or the prevalence of the MTHFR C677T genotype. Four articles were excluded because they had insufficient data regarding plasma tHcy levels, only reporting on the proportion of hyperhomocysteinemia (hyperhomocysteinemia defined as plasma tHcy >15 μmol/L). Two articles were excluded because they were cross-section studies. Two articles contained duplicated data and one article compared the plasma tHcy concentrations between single-episode CRVO patients and recurrent CRVO patients. Finally, 42 case–control studies were included in this meta-analysis [9, 10, 14, 17–20, 27–61]. The study selection process is shown in Figure 1.

### Study characteristics and quality assessment

All studies were case–control in design. Table 1 shows the studies identified and their main characteristics. The studies were published between 1998 and 2014, and they originated from the United States, Israel, Sweden, the United Kingdom, Ireland, Italy, Austria, Argentina, Saudi Arabia, France, Iran, Turkey, Thailand, China, India, and Brazil. In total, 2,794 cases and 3,651 controls were included in the meta-analysis. The controls were mainly healthy populations without retinal vascular disease. The NOS results showed that the average score was 7.11 (range 6–8), indicating that the methodological quality was generally good (Table 1).

**Table 1:** **Characteristics of enrolled case–control studies**

Author (year)	Country	Fasting	No. of RVO patients			No. of controls	Age (case/control, y)	Sex (case/control; M/F)	Source of cases	Source of controls	Matching	Reported Plasma tHcy concentrations or MTHFR C677T genotype	NOS score
			Total	CRVO	BRVO								
Salomon (1998) [59]	Israel	No	102	45	48	105	NA	58/44; 65/40	CP	Hospital patients with non-retinal vascular diagnosis	Age	MTHFR C677T	7
Glueck (1999) [25]	United States	No	17	NA	NA	234	52/37	8/9;NA	CP	“Healthy subjects”	NA	MTHFR C677T	6
Vine (2000 ) [26]	United States	No	74	74	0	74	69.8/64.6	29/45; 33/41	HR	Hospital patients with non-retinal vascular diagnosis	Age	tHcy	8
Larsson (2000) [27],^a^	Sweden	No	37	37	0	65	40.9/40.9	67/49; 110/30	HR	“Randomly selected”	Age	tHcy, MTHFR C677T	8
			79	79	0	88	69.6/69.6						
Pianka (2000) [28]	Israel	No	21	21	0	81	58.6/66	NA	CP	“Healthy adults”	Age, Sex	tHcy	6
Martin (2000) [9]	United Kingdom	Yes	60	36	24	85	65.6/51.5	NA	CP	Laboratory staff/hospital patients	NA	tHcy	7
Cahill (2000) [29]	Ireland	Yes	61	40	21	87	69.2/70.2	29/32; 36/51	HR	Hospital patients, primarily cataract extraction	Age	tHcy, MTHFR C677T	8
Boyd (2001) [30]	United Kingdom	No	63	63	0	63	60.3/60.8	NA	CP	Hospital patients with non-retinal vascular diagnosis	Age	tHcy, MTHFR C677T	8
Marcucci (2001) [31]	Italy	Yes	100	100	0	100	Median 59/ 56	54/46; 58/42	CP	Friends/partners, no cardiovascular disease	Age, Sex	tHcy, MTHFR C677T	7
Weger (2002) [32]	Austria	Yes	84	0	84	84	68.1/68.2	37/47; 37/47	CP	Hospital patients,	Age	tHcy, MTHFR C677T	8
Adamczuk (2002) [33]	Argentina	Yes	37	37	0	144	NA	17/20; 66/78	CP	“Volunteers”	Age, Sex	MTHFR C677T	7
Brown (2002) [34]	United States	Yes	20^b^	15	3	20	69.1/69.5	12/8; 10/10	HR	“Normal subjects”	Age, Sex	tHcy	8
Weger (2002) [35]	Austria	Yes	78	78	0	78	68.7/68.6	33/45; 33/45	HR	Hospital patients	Age, Sex	tHcy, MTHFR C677T	8
El-Asrar (2002) [36]	Saudi Arabia	Yes	48	36	12	59	45.3/46.1	NA;44/15	CP	“Healthy adults”	Age, Sex	tHcy	6
Blondel (2002) [58]	France	No	101	85	14	29	54/51.0	45/56; 13/16	CP	Source not given	Age	tHcy	7
Marcucci (2003) [37]	Italy	Yes	55	26	29	61	Median 57/ 56	24/31; 27/34	CP	Friends/partners,	Age, Sex	tHcy, MTHFR C677T	8
Parodi (2003) [38]	Italy	Yes	31	31	0	31	44.5/44.2	19/12; 19/12	CP	“Volunteers”	Age, Sex	tHcy, MTHFR C677T	7
Dodson (2003) [39]	United Kingdom	NA	40	NA	NA	40	Median 66.1/ 66	21/19; 21/19	CP	“healthy adults”	Age, Sex	MTHFR C677T	7
Yaghoubi (2004) [40]	Iran	Yes	24	10	14	24	61.1/61. 7	11/13; 12/12	CP	Hospital patients	Age	tHcy	6
Yildirim (2004) [41]	Turkey	Yes	33	9	20	25	61.0/58.0	15/18; 11/14	CP	NA	Age, Sex	tHcy	7
Atchaneeyas-akul (2005) [42]	Thailand	Yes	32	11	15	88	53.8/54.4	19/22; 41/49	CP	Volunteers	Age, Sex	tHcy	6
Ferrazzi (2005) [43]	Italy	Yes	69	NA	NA	50	64.1/58.4	40/29; 38/12	CP	Volunteers	Age	tHcy, MTHFR C677T	8
McGimpsey (2005) [44]	United Kingdom	No	106	60	46	98	67.9/68.4	55/51; 45/53	HR	Clinic patients /friends	Age, Sex	tHcy, MTHFR C677T	8
Gao(2006) [45]	China	Yes	64	64	0	64	59.5/59.5	33/31; 33/31	CP	Volunteers	Age, Sex,	tHcy, MTHFR C677T	7
Gumus (2006) [46]	Turkey	Yes	82	26	56	78	57.7/57.4	36/46; 33/45	CP	Patients with refractive errors, presbyopia, or cataract	Age, Sex	tHcy	7
Lattanzio (2006) [47]	Italy	Yes	58	58	0	103	39.8/40.3	38/20; 59/44	CP	Hospital staff	Age, Sex	tHcy	7
Pinna (2006) [48]	Italy	Yes	75	33	42	72	63.9/63.5	40/35; 37/35	CP	Friends/partners/hospital staff	Age, Sex	tHcy	8
Narayanasam-y (2007) [49]	India	Yes	29	29	0	57	31.0/27.0	22/7; 41/16	CP	Hospital staff/students	Age, Sex	tHcy	8
Biancardi (2007) [50]	Brazil	No	55	NA	NA	55	NA	23/32; 23/32	CP	Hospital patients	Age, Sex	MTHFR C677T	6
Moghimi (2008) [51]	Iran	Yes	54	54	0	51	59.8/63.0	32/22; 29/22	CP	Clinic patients	Age, Sex,	tHcy	7
Sofi(2010) [52]	Italy	Yes	262	NA	NA	262	Median 66.0/ 65.5	122/140; 123/139	CP	Healthy subjects	Age, Sex	tHcy	8
Di Capua (2010) [53]	Italy	Yes	117	NA	NA	202	54.0/52.0	61/56; 105/97	CP	Volunteers	Age, Sex	tHcy, MTHFR C677T	7
Pinna(2010) [54]	Italy	Yes	40	0	40	80	64.3/63.2	19/21; 38/42	CP	“Normal subjects”	Age, Sex	tHcy	8
Sottilotta (2010) [14]	Italy	No	105	17	88	226	58.4/55.7	46/59; 44/182	CP	Healthy participants	Age	MTHFR C677T	7
Pinna (2010) [55]	Italy	Yes	29	29	0	80	63.2/63.2	15/14; 38/42	CP	Healthy participants	Age, Sex	tHcy	6
Tea (2013) [19]	France	No	21	21	0	23	46/46	14/7;15/8	CP	Volunteers	Age, Sex	MTHFR C677T	7
Bharathi (2012) [56]	India	Yes	23	23	0	57	30.0/28.0	17/6; 38/16	CP	Volunteers	Age, Sex	tHcy	6
Dong (2013) [18]	China	Yes	68	68	0	68	58.6/58.6	28/40; 28/40	CP	Hospital patients	Age, Sex	tHcy, MTHFR C677T	7
Lahiri (2013) [10]	India	Yes	64	24	40	45	NA	NA	CP	NA	Age, Sex	tHcy	7
Minniti (2014) [17]	Italy	Yes	91	47	44	71	57/55	51/40; 30/41	HR	Volunteers	Age, Sex	tHcy, MTHFR C677T	7
Mrad(2014) [20]	Tunisia	Yes	72	NA	NA	140	48.5/51.7	50/22; 95/45	HR	Healthy participants	Age	tHcy, MTHFR C677T	7
Russo (2014) [57]	Italy	Yes	113	NA	NA	104	NA	57/56; 75/29	CP	Volunteer controls	Age, Sex	MTHFR C677T	6

^a^Data presented in 2 age groups: <50 years and >50 years.

^b^Includes others (e.g., hemi-retinal, hemispheric, macular).

RVO = retinal vein occlusion; CRVO = central retinal vein occlusion; BRVO = branch retinal vein occlusion; M = male; F = female; CP = consecutive patients; HR = Hospital records; NA = not available.

### Plasma tHcy level outcomes

**Figure 2:**
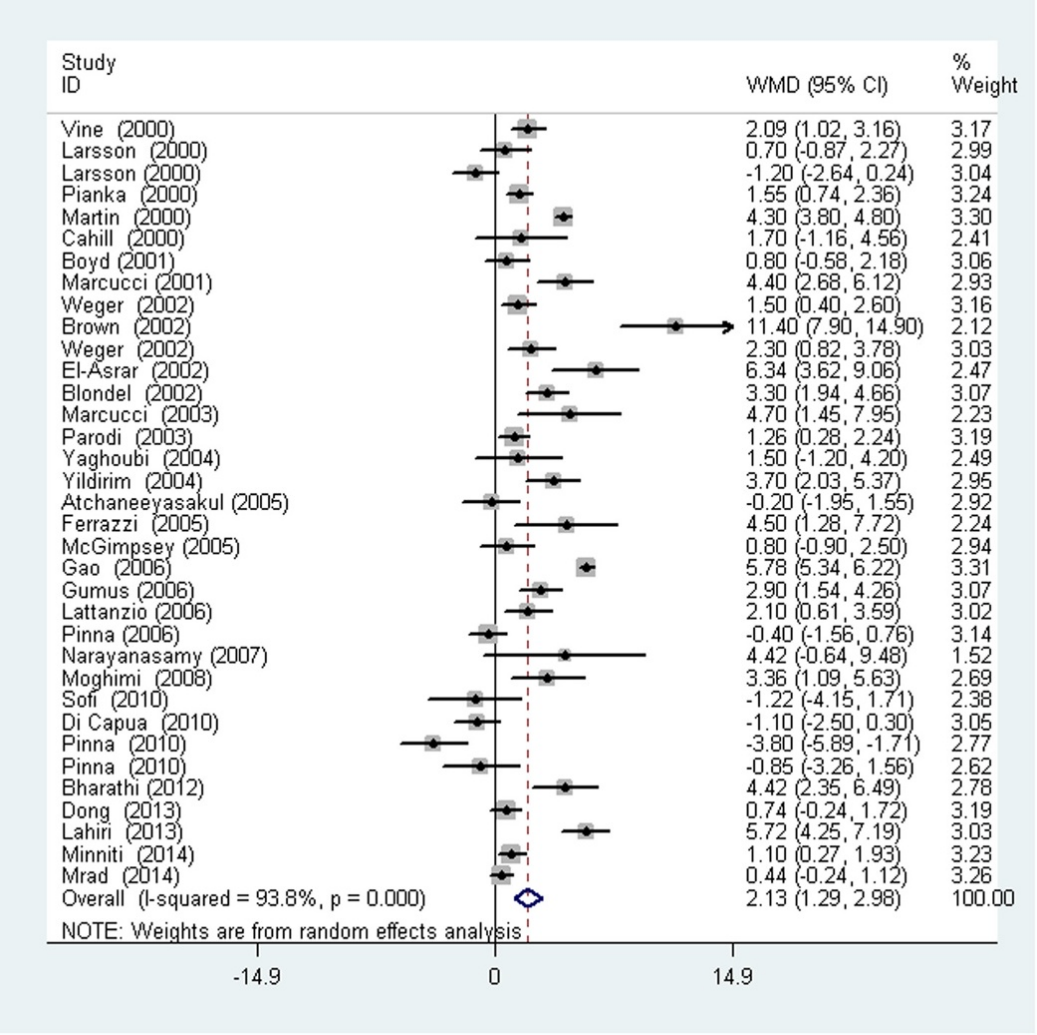
**Meta-analysis of the average plasma tHcy level of the RVO patients and controls.** *WMD* weighted mean difference, *CI* confidence interval. (Larsson et al. [29]): Data presented for two age groups: <50 years and >50 years.

**Figure 3:**
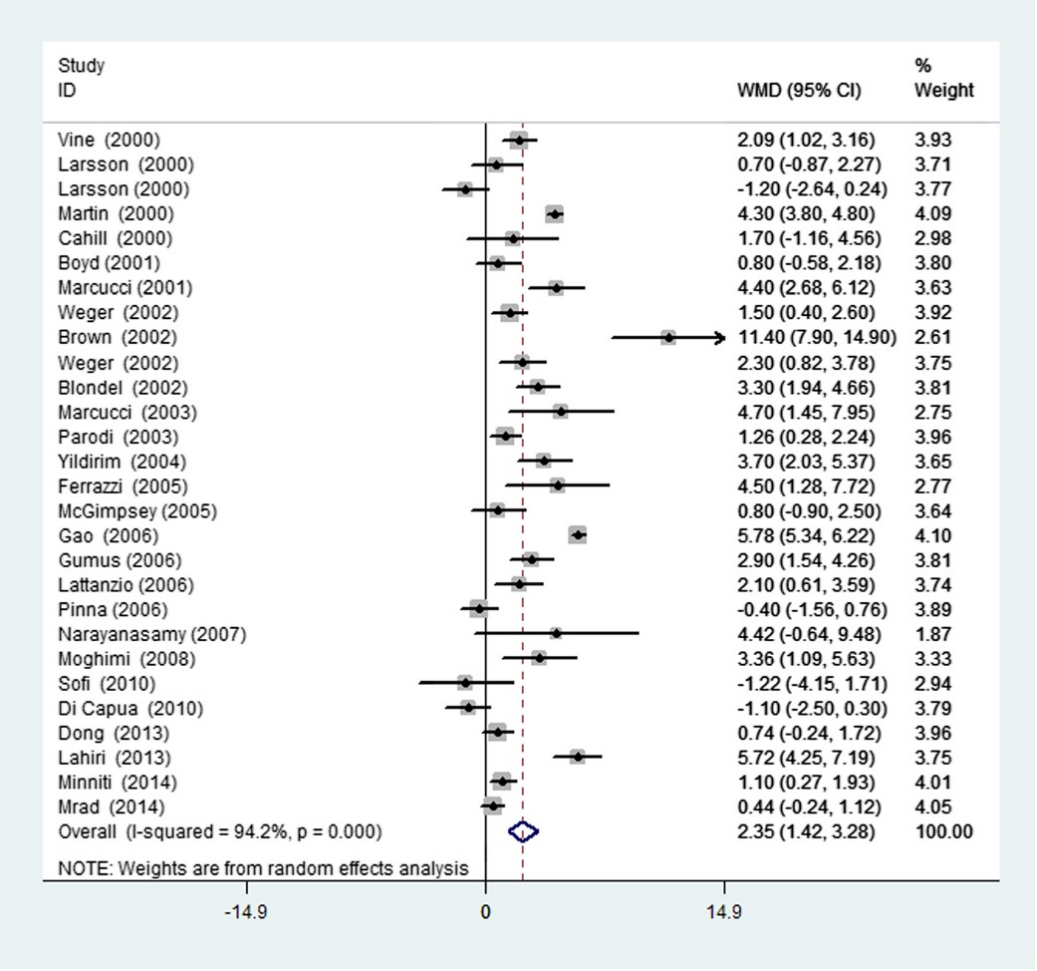
**Forest plot of the average plasma tHcy level of the RVO patients and controls after omitting the low-quality studies.** *WMD* weighted mean difference, *CI* confidence interval.

The analysis of the average plasma tHcy level of the RVO patients and controls in 34 studies revealed significant heterogeneity (I^2^ = 93.8%, *P <* 0.001) across the articles. Therefore, the data were pooled ina random-effects model. The meta-analysis of these data showed that the plasma tHcy level was significantly higher in the RVO patients than in the controls (WMD =2.13 μmol/L; 95% CI: 1.29–2.98, *P <* 0.001, Figure 2). Table 2 shows the detailed results stratified by the characteristics of the study. Overall, the plasma tHcy level was significantly higher in the RVO patients than in the control subjects, and this was consistently observed in each subgroup. Moreover, there was evidence of heterogeneity in all subgroups. Table 2 presents the results of the meta-regression analysis of the influence of the key characteristics of the studies (subgroup factors) on heterogeneity. After the exclusion of low-quality studies, the random-effects estimates were not changed substantially, suggesting a high stability of the meta-analysis results (WMD =2.35 μmol/L; 95% CI: 1.42–3.28, *P* < 0.001, Figure 3). With regard to the plasma tHcy level outcomes, Begg’s rank correlation test and Egger’s linear regression test provided little evidence of publication biases among the studies (Begg, *P* =0.091; Egger, *P* =0.051).

**Table 2:** **Subgroup analysis of pooled estimates for the mean plasma tHcy in the cases compared with the controls**

Subgroup	Studies (n)	WMD (95%CI)	Test for overall effect	Study heterogeneity			***P***for meta-regression
				χ ^2^	***P***	I ^2^	
Overnight fast							0.269
Yes	28	2.41 (1.41, 3.41)	Z =4.71, *P* <0.001	481.24	<0.001	94.4%	
No	7	1.20 (0.25, 2.16)	Z =2.46, *P* =0.014	23.70	0.001	74.7%	
Diagnosis							0.343
RVO^a^	18	2.56 (1.39, 3.72)	Z =4.31, *P* <0.001	222.86	<0.001	92.4%	
CRVO	17	1.67 (0.39, 3.00)	Z =2.55, *P* =0.011	314.88	<0.001	94.9%	
Source of cases							0.696
Hospital records	9	1.60 (0.47, 2.74)	Z =2.76, *P* =0.006	52.86	<0.001	84.9%	
Consecutive patients	26	2.24 (1.25, 3.23)	Z =4.44, *P* <0.001	401.13	<0.001	93.8%	
Adjusting factors							0.245
NA	1	4.30 (3.80, 4.80)	Z =17.00, P <0.001	-	-	-	
Age	10	1.33 (0.47, 2.18)	Z =3.05, P =0.002	32.23	<0.001	72.1%	
Age, sex	24	2.34 (1.19, 3.50)	Z =3.98, P <0.001	409.41	<0.001	94.4%	

^a^RVO subgroup includes CRVO, BRVO and others (e.g., hemi-retinal, hemispheric, macular).

tHcy = total homocysteine; WMD = weighted mean differences; CI = confidence interval; RVO = retinal vein occlusion; CRVO = central retinal vein occlusion.

### Association between plasma tHcy and RVO

**Figure 4:**
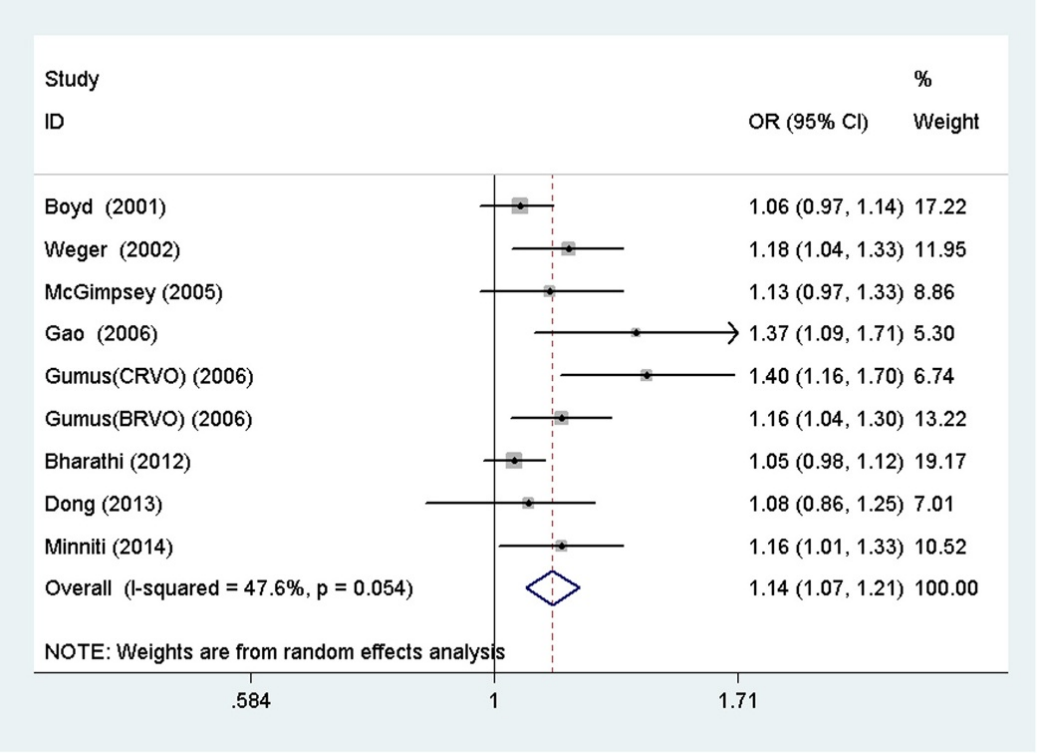
**Forest plot of the risk estimates of the association between plasma tHcy and RVO.** *OR* odds ratio, *CI* confidence interval.

We identified nine studies that reported an association between tHcy and RVO. As shown in Figure 4, a 1 μmol/L increase in the plasma tHcy level was associated with an OR of 1.14 (95% CI: 1.07–1.21) in the random-effects model, showing a statistically significant association between tHcy and the risk of RVO. The heterogeneity was statistically insignificant (I^2^ = 47.6%; *P* =0.054).

### Association between the MTHFR C677T genotype and RVO

**Figure 5:**
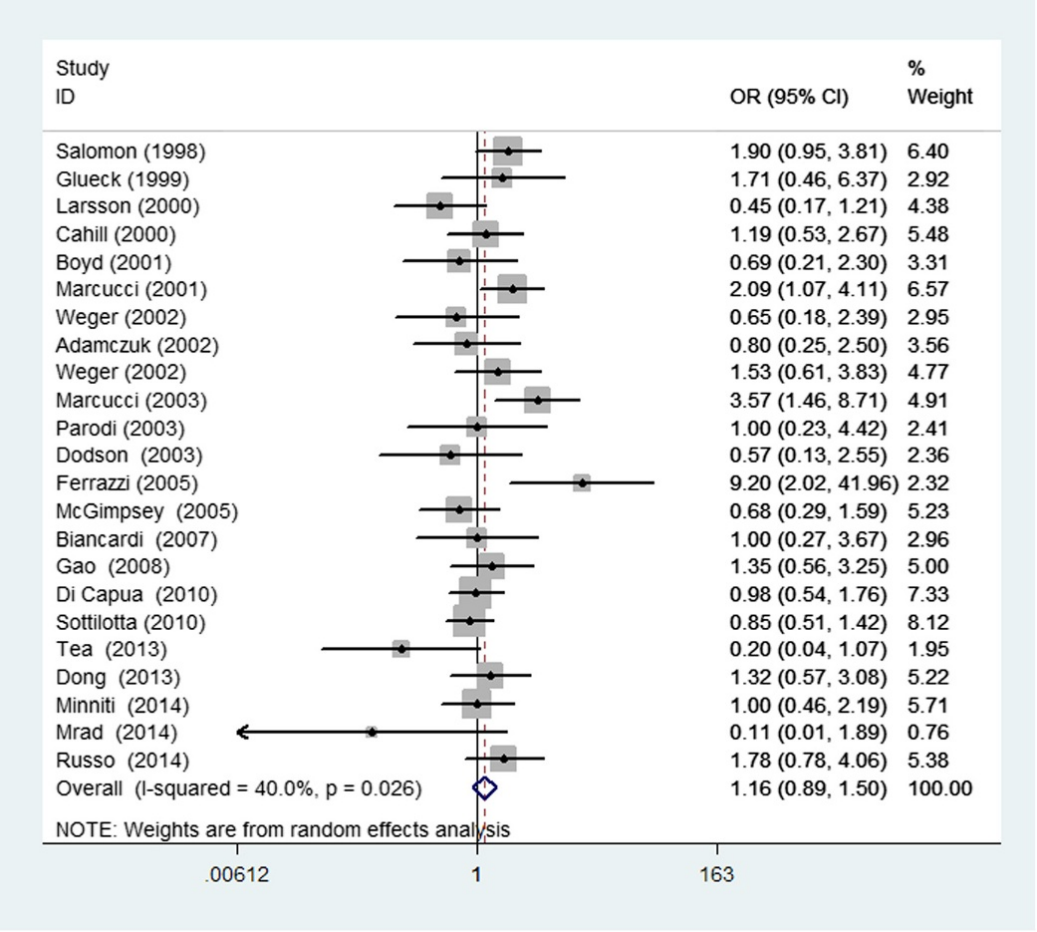
**Forest plot of the risk estimates of the association between the MTHFR C677T genotype and RVO (recessive model, TT vs. TC/CC).** *OR* odds ratio, *CI* confidence interval.

The pooled ORs with their respective 95% CIs and the result of the heterogeneity test are presented in Table 3 and Figure 5. Overall, there was no evidence of a significant association between the MTHFR C677T genotype and RVO in any genetic model tested (TT VS. CC/CT: OR = 1.16, 95% CI =0.89–1.50; CC VS. TT/CT: OR = 1.02, 95% CI =0.73–1.41; TT VS. CC: OR = 1.30, 95% CI =0.85–1.98; CT VS. CC: OR = 1.22, 95% CI = 0.90–1.66). The I^2^ statistic indicated substantial between-study heterogeneity in all genetic models tested. For MTHFR, the Begg’s test and Egger’s test also showed little evidence of publication biases among the studies (Table 3).

**Table 3:** **Analyses of the MTHFR C677T genotype and RVO**

Compared genotype	No. of studies	OR (95% CI)	***P***	Heterogeneity			***P***Egger’s test ^a^	***P***Begg’s test ^b^
				x ^2^	I ^2^	***P***		
TT VS. CC/CT	23	1.16 (0.89–1.50)	0.268	36.66	40.0%	0.026	0.551	1.000
CC VS. TT/CT	14	0.77 (0.57,1.05)	0.098	37.78	65.6%	<0.001	0.510	0.584
TT VS. CC	14	1.30 (0.85,1.98)	0.223	28.78	54.8%	0.007	0.056	0.063
CT VS. CC	14	1.22 (0.90,1.66)	0.202	34.8	62.6%	0.001	0.109	0.101

^a^ *P* Egger’s test = the *P* value for Egger’s test.

^b^ *P* Begg’s test = the *P* value for Begg’s test.

## Discussion

The present meta-analysis evaluated the relationship among plasma tHcy, the MTHFR C677Tgenotype, and RVO, and it included only case–control studies. The data provide a greater ability to assess the potential correlation between the aforementioned factors. We combined the effect sizes of 34 studies, which compared plasma tHcy levels between RVO patients and controls, in a random-effects model. The results demonstrated that the plasma tHcy level was significantly higher in the RVO patients than in the controls, with a pooled WMD of 2.13 μmol/L (95% CI: 1.29–2.98). A meta-analysis of data collected before September 2009revealed that the mean tHcy in the cases was 2.8 μmol/L (95% CI: 1.8–3.7) greater than in the controls [15]. Our findings are consistent with those of the earlier meta-analysis. Of note, when we analyzed the association between plasma tHcy and RVO, we found that a 1 μmol/L increase in the plasma tHcy level was associated with an OR of 1.14. Moreover, in the present meta-analysis, in an attempt to produce robust results, we performed subgroup and sensitivity analyses based on various characteristics of the study. The results of the subgroup and sensitivity analyses did not materially alter the pooled results, thereby supporting the robustness of our main finding. The possible mechanisms by which tHcy may contribute to RVO include the activation of factor V, the increased oxidation of low-density lipoprotein, the inhibition of plasminogen activator binding, and the activation of protein C [62].

The previous meta-analysis investigated the association between the MTHFR C677T genotype and RVO and found no association between the homozygosity of the TT genotype or RVO. The authors speculated that one possible cause of this lack of association was the modest number of studies included in the meta-analysis. However, with the added statistical power of 1,682 cases, the present meta-analysis also found no significant association between the MTHFR C677T genotype and RVO under all genetic models. Genetic factors are not the only factors capable of increasing the tHcy level; demographic and lifestyle factors, such as age, gender, folate intake, smoking status, vitamin B levels, systemic vascular diseases, and use of antihypertensive medications, can affect the plasma tHcy level [63].

The present meta-analysis identified substantial heterogeneity among the studies. This was not surprising, given the differences in the characteristics of the populations, data collection methods, ethnic populations, sample size, and sources of the cases. Whenever significant heterogeneity was present, a subgroup analysis was conducted, and a random-effects model was used to pool the results. However, our attempts to identify homogeneous subsets largely failed in the subgroup analysis, with heterogeneity remaining in all the subgroups in the studies. The meta-regression analysis also failed to identify the main sources of the heterogeneity. Several factors might account for the heterogeneity. First, environmental exposure and diet might play roles [63]. Second, some unpublished, eligible publications were unavailable for inclusion in the present meta-analysis, and this might have affected the results. Thus, the results should be considered with caution.

The previous meta-analysis analyzed data from 25 case–control studies and found that plasma tHcy level was relatively higher in RVO patients compared with controls [15]. The authors also found no association between the MTHFR C677T genotype and RVO. However, the meta-analysis contained a number of weaknesses. First, the authors reported the difference in the plasma tHcy level between the cases and controls, but not the dose-effect relationship between tHcy and RVO. In the present meta-analysis, we found that a 1 μmol/L increase in the plasma tHcy level was associated with an OR of 1.14. In addition, the previous meta-analysis did not have rigorous inclusion criteria [15]. For example, they included a case series study, and they only indirectly compared the cases and controls [64].

The results of the present meta-analysis must be interpreted cautiously in light of the strengths and limitations of the included studies. A major strength of this study is the enlarged sample size, as compared to the previous meta-analysis, and we added 17 newly published case–control studies, which provides enhanced statistical power and offers more precise and reliable effect estimates. Furthermore, we only included the case–control studies and no other studies. In addition, the methodological issues for the meta-analysis, such as publication bias and the stability of results, were well investigated. Our meta-analysis also has several limitations. One potential limitation is the substantial heterogeneity observed among the studies. Second, the case–control study design means that the assessment of tHcy in patients at varying time intervals after the occlusive vascular event is methodologically weak. The vascular occlusive event itself could increase the tHcy concentration. Third, to avoid publication bias, we performed not only an electronic search but also a manual search to identify all potentially relevant papers, including published and non-published sources. Unfortunately, we may have failed to include some papers, especially those published in other languages. Publication bias may have resulted in an overestimate of the relationship between tHcy and RVO. Fourth, in some studies, age was not entirely matched between the case and control groups. There is some evidence that tHcy increases with age, which might have affected the pooled results. Fifth, the controls were not uniformly defined. This was a meta-analysis of case–control studies, and no studies were population-based. Thus, some inevitable selection biases might exist in the results, and they may not be representative of the general population.

## Conclusions

In conclusion, despite these limitations, the current meta-analysis of observational studies suggests that an elevated level of plasma tHcy increases the risk of RVO. There was no evidence to suggest an association between the MTHFR C677T genotype and RVO. Despite these encouraging findings, the inherent limitations of the included studies should be considered, and conclusions drawn from our pooled results should be interpreted with caution.
